# A review of recommendations, efficacy, and patient safety for over-the-counter norgestrel for daily contraception

**DOI:** 10.1016/j.rcsop.2025.100591

**Published:** 2025-03-11

**Authors:** Gia Tran, Joshua Wollen, Shantera Davis, Elisabeth M. Wang, Julia Arriazola, Natalie Rosario

**Affiliations:** a University of Houston College of Pharmacy, United States of America; b Pharmacy Practice and Translational Research, University of Houston College of Pharmacy, 4349 Martin Luther King Boulevard,Houston, TX 77204-5039, United States of America; c Dept. of Pharmacy Practice and Translational Research, University of Houston College of Pharmacy, 4349 Martin Luther King Boulevard | Office 4011, Houston, TX 77204, United States of America

**Keywords:** Opill, Progestin-only pill, Contraception, Contraceptive access, Over-the-counter

## Abstract

**Purpose:**

This narrative review examines the implications of norgestrel, a progestin-only oral contraceptive, becoming newly available over-the-counter (OTC) in the United States. The objectives are to explore the pharmacotherapy,mechanism of action, efficacy, safety, and implications to clinical practice of OTC progestin-only pills (POPs), and how this may impact contraceptive access and public health.

**Methods:**

The review synthesizes data from clinical studies, public health reports, global perspectives, and recent policy changes to assess the potential impact of OTC access to norgestrel in the United States. The analysis includes the evaluation of the pharmacodynamics of norgestrel 0.075 mg on ovarian activity, the effectiveness of POPs, and benefits and barriers to contraceptive access.

**Findings:**

The evidence indicates that POPs thickens cervical mucus, providing effective contraception within 48 h of use. While ovulation inhibition may take up to 28 days and occurs in approximately half of users, the availability of POPs OTC is anticipated to significantly reduce barriers to contraceptive access. This could lead to a broader use of effective contraception and potentially reduce unintended pregnancies. Additionally, the review highlights that the introduction of OTC contraceptives could increase access among populations historically facing difficulties in obtaining prescriptions.

**Conclusions:**

Making norgestrel available OTC represents a critical advancement in contraceptive access with the potential to enhance public health by reducing unintended pregnancies. However, the extent of its impact will depend on widespread education and adherence to proper use. The review underscores the need for further research to monitor outcomes post-OTC availability and to assess the broader implications for reproductive health and equity.

## Highlights

• Many countries have hormonal contraceptives available without a prescription.
• The US approved over-the-counter norgestrel, a progestin only contraceptive.
• Access to contraceptives can improve childbearing individuals' quality of life.
• Progestin only contraceptives are generally safe and efficacious.

## 1 Introduction

Oral contraceptives have significantly impacted reproductive health since their introduction in the United States (US) in the 1960s.^1^ While the methods of oral contraceptive studies and US Food and Drug Administration (FDA) approval relied on lack of informed consent of Puerto Rican women, the impact of the studies have been long-lasting.2., 3., 4., 5. The availability of birth control has contributed to significant advancements in women's education and employment in the US. In the early 1970s, some states allowed unmarried women to access the pill at age 18 instead of 21 leading to notable improvements in access. Women in states with earlier and easier access to the pill were 10 % to 20 % more likely to be enrolled in college by age 21 and experienced higher earning trajectories that lasted into their 40s. These benefits persisted even when considering other factors, demonstrating the substantial impact of increased contraceptive access on women's socioeconomic outcomes.^6^

Estimates from the Centers for Disease Control and Prevention National Center for Health Statistics show significant declines in both overall and unintended pregnancy rates in the US from 2010 to 2019. Additionally, pregnancy rates and unintended pregnancy rates for teens aged 15–19 decreased by more than half (52 %) during this period. Notable declines in unintended pregnancy rates were observed across different demographic groups: 23 % among Hispanic females, 17 % among non-Hispanic females of races other than Black or White, 12 % among Black non-Hispanic females, and 11 % among White non-Hispanic females.^7^ While increased access to contraception is a factor in the overall decline in pregnancies, it is one of several contributing elements in addition to public health interventions aimed at reducing unintended pregnancies. Other factors that likely contribute to this trend include economic conditions such as inflation (which may influence family planning decisions), increased health literacy and awareness about reproductive health, and a possible shift in societal attitudes and desires regarding having children. These multifaceted influences highlight the complexity of understanding and addressing changes in pregnancy rates.

In many countries, oral contraceptives require a prescription, limiting their accessibility and posing a significant barrier to consistent and effective contraceptive use.^8^, ^9^, ^10^ In a publication by Grindlay et al. (2023), countries were classified into four categories regarding the status of oral contraceptive access: 1) informally available without prescription, where a prescription is legally required but often not enforced; 2) legally available without prescription and no required health screenings; 3) legally available without prescription but with required health screenings by a professional; and 4) available only with a prescription.^11^ This classification provides a comprehensive overview of the varying degrees of accessibility worldwide.^12^ There has been a notable increase in access to over-the-counter (OTC) oral contraceptives globally. According to Free the Pill, a nonprofit organization, there are more than 100 countries worldwide who have an OTC birth control pill available.^12^ Free the Pill notes that this trend reflects ongoing efforts to reduce barriers to contraceptive access and enhance reproductive autonomy worldwide.

The Contraceptive CHOICE Project, one of the largest prospective cohort studies of participants in the US seeking reversible contraception, found that when barriers such as cost, access, and knowledge are removed, they continued using these methods with high satisfaction. As demonstrated by the St. Louis CHOICE project, where participants who received contraceptive counseling, a free method of their choice, and a patient-centered clinic experience had an abortion rate less than half that of nonparticipants in the same area and roughly one-fourth the national rate.^13^ Considerations of contraception use vary, with many users prioritizing their preferences according to effectiveness, reliability, ease of use, cost, discreet use, and side effect and safety profile.^14^ Oral contraceptives are 99 % effective at preventing pregnancy when taken as prescribed and without interacting medications. However, according to a review by Teal in 2021, typical use in the US accounting for human error such as missing doses, found a pregnancy rate of 17.8 % in patients using oral contraception.^15^

The expansion of OTC availability signifies progress in making effective contraceptive options more readily accessible, contributing to improved public health outcomes and greater reproductive autonomy.^3^^,^^12^ The FDA approval of Opill®, a progestin-only oral contraceptive pill containing norgestrel 0.075 mg, in the US as an OTC option marks a significant milestone in this progression. This FDA approval not only allows easier access but also represents a shift towards recognizing the importance of reproductive health care accessibility in the US. This review explores the implications of OTC norgestrel's approval, focusing on the pharmacology, recommendations, efficacy, and patient safety. Through a comprehensive examination of current literature and clinical data, this review will provide an in-depth understanding of how OTC norgestrel fits into the evolving landscape of contraceptive options and what its availability means for future contraceptive practices and policies.

## 2 Methods

### 2.1 Data selection

A literature search was performed using the following search terms: *Norgestrel 0.075* *mg*, *Opill, over-the-counter, OTC,* and *contraception.* Abstracts, product monographs, randomized controlled trials, cohort studies, narrative reviews, and systematic reviews in human subjects published in English were reviewed for potential inclusion for this narrative review. Studies were excluded if they solely discussed *non-oral progestin dosage forms, combined oral contraceptive (COC), emergency contraception,* or *non-contraceptive use of progestins*. Databases included PubMed and the Institutes of Health Clinical Trials Registry (http://www.clinicaltrials.gov). The search filter ranged from January 1, 2010, to May 22, 2024. Articles were reviewed and included if they contained information on progestin-only oral contraceptives available OTC. Additionally, literature regarding the availability and accessibility of OTC oral contraception in other countries, such as those in Asia, Europe, and Mexico, was specifically sought to provide a comprehensive global perspective. This approach ensured a thorough understanding of the international landscape of OTC contraceptive availability and its implications for safety, efficacy, adherence and tolerability.

### 2.2 Global perspective

The availability and accessibility of OTC oral contraceptives vary widely across different countries, reflecting internationally diverse regulatory environments and healthcare systems. Select examples are described to highlight the variability in global access to OTC norgestrel. For example, in England, a significant step was taken in November 2023 to enhance contraceptive access where patients can now obtain contraceptive pills directly from pharmacies without needing a referral from their general practitioner.^16^ This initiative is part of England's National Health Service (NHS) primary care access recovery plan, designed to make healthcare more accessible. Pharmacists, after undergoing training, can now offer confidential consultations and supply contraceptive pills, which significantly reduces the barriers to obtaining contraception.^16^ This includes combined oral contraceptives or progestin-only contraceptive pills.^17^

Due to a variety of policies in different countries, it can be complex to determine what is available and accessible for OTC contraception. Vietnam has further improved contraceptive access through the Population and Family Health Project, which emphasizes the importance of accessibility. This project aimed to strengthen primary healthcare systems in provinces with low contraceptive prevalence and high unintended pregnancy rates by making contraceptives, including oral contraceptives, available OTC at local health clinics and drugstores. The initiative significantly increased contraceptive use, highlighting how increased access supports reproductive health goals.^18^

In Mexico, OTC access to oral contraceptives has been available for a considerable time and has significantly impacted contraceptive use patterns. According to a national survey conducted in 2014, 54 % of pill users, including a substantial 66 % of adolescents aged 15–17, obtained their oral contraceptives OTC. This survey also highlighted that those without insurance and those living in urban areas were more likely to use OTC contraceptives.^19^ The availability of contraceptives without the need for a prescription has played a crucial role in enhancing access, especially for young and uninsured patients, who may face more significant barriers in accessing healthcare services leading to unintended pregnancies.

## 3 Mechanism of action

Progestin-only oral contraceptives containing norgestrel prevent pregnancy through multiple mechanisms of action. One mechanism is the suppression of ovulation, which occurs in approximately half of users.^20^ By lowering the midcycle peaks of luteinizing hormone and follicle-stimulating hormone, the pill inhibits the release of an egg from the ovaries, thereby preventing ovulation. Progestin-only pills (POPs) also disturb follicle growth, which can prevent the full development of ovarian follicles and contributes to their contraceptive efficacy. POPs slow down the movement of the ovum through the fallopian tubes, further decreasing the chances of fertilization.^20^^,^^21^ Additionally, POPs thicken the cervical mucus, creating a barrier that significantly impedes sperm penetration through the uterus, thereby reducing the chances of fertilization. Norgestrel also alters the endometrium, the lining of the uterus, making it less suitable for implantation.^22^ Overall, the combination of ovulation inhibition, disturbance of follicle growth, delayed ovum movement, thickening of cervical mucus, and alteration of the endometrium ensures that POPs are highly effective in preventing pregnancy.

## 4 Pharmacokinetics

Norgestrel is rapidly absorbed and eliminated. Approximately two hours after oral administration, serum progestin levels peak, followed by rapid distribution throughout the body and subsequent elimination. At 24 h post-ingestion, serum levels of progestin are near baseline, necessitating strict adherence to the dosing schedule to maintain contraceptive efficacy. Progestin-only administration results in lower steady-state progestin levels and a shorter elimination half-life compared to when progestins are administered with estrogens. Additionally, there is significant variability in serum progestin levels among individual users, which can influence the overall effectiveness and side effect profile of the contraceptive. This variability necessitates careful consideration and the importance of the same time daily administration schedule to ensure optimal contraceptive protection.^21^

## 5 Dosing and administration

OTC norgestrel is administered as a daily oral contraceptive. The tablets are packaged in a 28-day blister pack, and users should take one tablet orally at the same time every day without any breaks between packs. For optimal effectiveness, the POP should be taken at the same time every day (ie: 8 AM), or taking into account real-world unintentional late doses no later than three hours of the previous day's dose (ie: 8–11 AM). If a dose is missed, the missed dose should be taken as soon as possible. Users should continue with their regular dosing schedule and practice abstinence or use a non-hormonal barrier contraceptive method, such as condoms, for the next 48 h to ensure contraceptive effects.^19^^,^^29^

## 6 Efficacy

Norgestrel has demonstrated high efficacy in preventing pregnancy. With typical use, POPs have a 91 % effectiveness rate, meaning up to 9 out of 100 users may become pregnant during the first year. However, with perfect use as directed, the effectiveness rate increases to 99 %, with fewer than 1 in 100 users becoming pregnant each year. Historical studies have also contributed to the understanding of POPs efficacy. Research from the 1970s and 1980s, although less rigorous by modern standards, consistently showed that POPs were effective in preventing pregnancy.^23^ For example, a study by Tejuja et al. (1974) in India demonstrated that both 0.050 mg and 0.075 mg/dl-norgestrel mini-pills were effective contraceptive options, with low pregnancy rates observed among users.^24^

### 6.1 Norgestrel Pearl Index rating

A comprehensive review of the literature, including multiple national and international studies, supports POPs effectiveness with a failure rate of approximately 2 per 100 person-years, which is comparable to other hormonal birth control methods.^23^^,^^25^ These studies collectively evaluated the pill's performance across various populations, in both breastfeeding and non-breastfeeding individuals. In non-breastfeeding individuals, six studies involving 3184 participants reported an overall Pearl Index (PI) of 2.2 during typical use, indicating that norgestrel 0.075 mg/day is effective in real-world conditions.^26^ The PI is a measurement of the efficacy of contraceptives calculated by dividing the number of pregnancies (numerator) by the number of years of exposure to the contraceptive (denominator) multiplied by 1200 if the denominator is measured in months, or 1300 if the denominator is measured in cycles.^27^ A lower PI indicates a more effective contraceptive. Another study included 4088 breastfeeding individuals and found a cumulative life table pregnancy rate of 1.2 per 100 person-years, suggesting similar effectiveness in this group.^26^

These studies highlight the importance of adherence to a strict dosing schedule due to the rapid elimination of norgestrel from the body, which requires the pill to be taken at the same time every day to maintain optimal efficacy. OTC norgestrel is effective 48 h after the patient begins taking it. Important patient counseling points include recommending that either abstinence or a barrier method be used for the first two days to help prevent unplanned pregnancy. If one or more days of OTC norgestrel is missed or if it is beyond three or more hours late taking a tablet, the patient should use a barrier method for 48 h upon restarting the regimen. The consistent use of norgestrel 0.075 mg tablets results in significant contraceptive protection, with some variability in serum levels among individual users. Despite these variations, the aggregate data indicate that POPs like OTC norgestrel are a highly reliable method of contraception when used correctly.^21^ This high level of efficacy, combined with the convenience of OTC availability, makes OTC norgestrel a valuable option for many individuals seeking effective birth control.

### 6.2 Evaluation of norgestrel on ovarian activity

A study conducted to understand the pharmacodynamics of norgestrel 0.075 mg focused on its effect on ovarian activity. This prospective, two-site, randomized, crossover study monitored ovarian activity using hormone measurements and ovarian ultrasonography. The study found that during 28 days of correct use, 66.6 % of participants exhibited no evidence of ovulation.^28^ These results highlight the importance of ovulation inhibition and follicle growth disturbance with norgestrel POPs.^29^ In real-world scenarios, it is important to note that while the POP begins to thicken cervical mucus within 48 h, it may take up to 28 days of consistent use to fully inhibit ovulation. However, ovulation inhibition occurs in only about half of the users.^23^ This dual mechanism emphasizes the importance of patient counseling. Healthcare providers should inform users about the immediate thickening effect on cervical mucus and the potential of gradual suppression of ovulation. This counseling point allows users to understand how to use the pill effectively to prevent unintended pregnancies during the initial phase of use.

### 6.3 Effect of deliberate non-adherence to norgestrel

Real-world efficacy of POP contraceptives is described in a prospective, two-site, randomized, crossover study that assessed the impact of intentional non-adherence to a norgestrel POP. This pragmatic approach considers unintentional late or missed doses of POP which may occur with typical use of contraceptives compared to the pharmacokinetic efficacy studies. Researchers monitored ovarian activity and cervical mucus scores in individuals who occasionally missed or delayed taking their pills. The results indicated that occasional missed or delayed doses did not significantly compromise the contraceptive efficacy. Most participants maintained cervical mucus scores unfavorable for sperm penetration, and ovulation rates remained low. This suggests that the pill remains effective even with minor lapses in adherence, making it a robust option for contraception.^20^

## 7 Safety

### 7.1 Adverse events and precautions

Common adverse events associated with OTC norgestrel include menstrual irregularities, such as changes in menstrual flow, spotting, and amenorrhea. Other reported side effects are headaches, dizziness, nausea, increased appetite, abdominal pain, cramps, bloating, backache, and breast discomfort.^21^ Users may also experience ovarian cysts, which are generally asymptomatic and resolve on their own but can occasionally cause pain and may require surgical intervention. Additionally, OTC norgestrel contains FD&C Yellow No. 5, which can cause allergic reactions in some individuals, particularly those with aspirin sensitivity.^21^

Those with medical conditions associated with higher risk of thromboembolic events or who are age 35 and use tobacco products usually will not be prescribed an estrogen-containing contraceptive due to increased clot risk. In contrast, POPs do not contain estrogen and may be a safer alternative. A systematic review showed that POPs did not increase the odds of thromboembolic events, stroke, or acute myocardial infarction.^30^ This supports the safe use of POPs by the general population as an OTC contraceptive.^31^

Additionally, progestin-only emergency contraceptives (ie: levonorgestrel) are available without a prescription in the US Based on the Yuzpe method, or taking multiple doses of a contraceptive pill as a form of emergency contraceptive, patients may use OTC norgestrel as an emergency contraceptive option despite not having an FDA approved indication for emergency contraception.^32^, ^33^, ^34^ Clinicians should be prepared to provide appropriate counseling on the safety and efficacy of available OTC POPs and emergency contraceptives given the increased access to norgestrel and how it compares to the FDA approved OTC emergency contraceptive, levonorgestrel.

### 7.2 Drug interactions

The effectiveness of OTC norgestrel may be reduced by hepatic enzyme-inducing drugs such as phenytoin, carbamazepine, barbiturates, rifampin, efavirenz, bosentan, and herbal products containing St. John's Wort. This could result in unintended pregnancy or breakthrough bleeding. During the use of these substances, a non-hormonal backup method of contraception is recommended for 28 days after discontinuation of the enzyme-inducing agent. Patients receiving long-term therapy with hepatic enzyme inducers should consider another method of contraception.

Tirzepatide, a dual glucagon-like peptide-1 and glucose dependent insulinotropic polypeptide, may decrease the oral absorption of hormonal contraceptives via the delayed gastric emptying effects.^35^ To prevent unintended pregnancies, the manufacturer recommends patients should be counseled on abstinence or a backup barrier method for four weeks after tirzepatide initiation and for four weeks after each tirzepatide dose escalation.^36^

Additionally, OTC norgestrel should not be used within five days of taking ulipristal acetate, an emergency contraceptive, as it may decrease the effectiveness of both medications. Users should be advised to consult a pharmacist, health care provider, or the product information of concomitant medications for potential interactions.^21^

### 7.3 Special populations

OTC norgestrel is suitable for use in post menarche patients. While there is no age restriction for purchase, the manufacturer has established the safety and efficacy in those age 15 and older.^21^ Because OTC norgestrel is a progestin-only, estrogen-free contraceptive pill, it is suitable for most people of reproductive age, including those who are breastfeeding, smoke tobacco products, or those who have a history of migraine with aura. However, OTC norgestrel is contraindicated in pregnant patients, individuals with a history of breast cancer or other progestin-sensitive cancers, those with liver disease or tumors, those with undiagnosed abnormal uterine bleeding, those with hypersensitivity to any component of this product, when used concomitantly with another hormonal contraceptive, and in patients whose sex assigned at birth was male. While OTC norgestrel has not been studied in postmenopausal individuals and is not indicated for this population, its safety and efficacy are well-established in the intended user groups.^21^^,^^22^^,^^37^

## 8 Cost and availability

As of March 2024, there are currently no age restrictions surrounding the purchase of OTC norgestrel. OTC norgestrel is widely available online and in stores across all states in the US. Consumers can purchase Opill® through various platforms including the official website, www.Opill.com, major online retailers, and national pharmacy retailers. The cost for a one-month supply, which consists of a 28-pill pack, ranges between $15 USD and $20 USD. For a three-month supply (84-pill pack), the price is approximately $50 USD as of May 2024.

## 9 Relevance to patient care and clinical practice

Several clinical trials have been conducted to evaluate the effectiveness, adherence, and user experience of progestin-only oral contraception. These trials provide a comprehensive understanding of how these contraceptives perform in both clinical settings and real-world scenarios. High efficacy, adherence rates, and positive user experiences are associated with norgestrel POPs as described below. OTC availability of norgestrel improves access and adherence among users.

### 9.1 Systematic review of OTC availability

A systematic review examined the impact of oral contraceptives available OTC on user experiences. The review included studies comparing OTC availability to prescription-only access, finding higher continuation rates among OTC users, and indicating that OTC availability could increase access and reduce unintended pregnancies.^38^ Users reported high patient satisfaction and users favoring OTC access. The systematic review highlights the support for OTC availability among patients and pharmacists, though less so among physicians with safety being the primary concern.^38^

The availability of OTC norgestrel may carry substantial public health benefits. Eliminating the need for a prescription for POPs in the US is expected to significantly reduce barriers to contraceptive access. This is particularly beneficial for individuals who encounter difficulties in obtaining a prescription, such as those with limited healthcare access, younger patients, and individuals without health insurance. The shift of norgestrel from prescription only to include OTC availability allows individuals to take greater control over their reproductive health, contributing to improved health outcomes and greater reproductive autonomy.

### 9.2 Adherence with continuous dose oral contraceptive: evaluation of self-selection and use (ACCESS) study

The ACCESS study was designed to evaluate whether consumers can adhere to the regimen for a POP (norgestrel 0.075 mg) when obtained OTC. This prospective, multicenter study involved 883 participants who reported using the pill in a simulated OTC environment for 24 weeks. Participants included 200 adolescents and 120 people who were identified as having low health literacy (less than 8th grade reading level or score of <60 on the validated Rapid Estimate of Adult Health Literacy in Medicine or Rapid Estimate of Adolescent Literacy in Medicine tool).^39^ The study found that 85 % of participants reported taking the pill correctly on 85 % or more of the days, and 96 % took their dose within three hours of the scheduled time. These high adherence rates suggest that POPs can be effectively used without direct healthcare provider intervention, highlighting the potential for OTC availability to improve contraceptive access and use.^39^ Given the favorable safety profile of norgestrel, consumers including adolescents and those with low health literacy showed appropriate use of OTC norgestrel based on the OTC labeling.

### 9.3 ACCESS follow up: real world user experience

A cross-sectional survey conducted from January 2020 to September 2021 evaluated the experiences of the ACCESS study users taking OTC POPs in the US to gather insights into their experience with use of simulated OTC norgestrel.^40^ This survey included 550 adult and 115 adolescent participants. Most (80 %) participants felt their menstrual bleeding was acceptable. Additionally, 77 % of participants who had used contraception in the month prior to the trial reported that their overall experience with the OTC POP was similar or better than with previous methods. Adult participants preferred getting OTC POP information from a webpage or app; however, adolescents preferred asking questions at the pharmacy followed by asking a physician or other healthcare provider.^40^ This highlights the critical role pharmacists can play in adolescent patient education regarding OTC norgestrel and contraceptive practices overall. Key benefits reported included less worry about pregnancy, easy access, fewer side effects, and greater autonomy in decision-making.^40^ These findings highlight the positive user experience and potential benefits of making norgestrel available OTC.

## 10 Conclusion

The approval of norgestrel as an OTC oral contraceptive marks a pivotal advancement in reproductive health in the US. OTC norgestrel offers a comparatively safe, effective, and convenient option for OTC oral contraception. This transition is poised to enhance accessibility, particularly for individuals who face barriers in obtaining prescriptions, thereby promoting broader use of effective contraception. The increased availability of OTC oral contraceptives including POPs demonstrates the potential benefits of OTC access in reducing these barriers, increasing adherence, and providing individuals with greater autonomy over their reproductive health decisions.^28^ The comprehensive evaluation of OTC norgestrel's pharmacology, efficacy, safety, and real-world application underscores its abilities as a reliable contraceptive option.

Furthermore, the global perspective on OTC contraceptive availability highlights the diverse approaches and impacts observed in various countries, reinforcing the potential benefits of such accessibility if implemented on a global scale. These examples from different countries illustrate the diverse approaches to OTC oral contraceptive access and their impacts on contraceptive use on a global scale. By reducing the need for prescriptions and making POP contraceptives more readily available, these initiatives have the potential of improving adherence, reduce unintended pregnancies, and empower individuals to have reproductive autonomy.

As healthcare adapts to this shift, it is essential to ensure affordability and proper education to maximize the public health benefits of OTC contraceptives. Future research evaluating outcomes of the availability of OTC norgestrel in the US will better help improve population health and policy, both within and outside of the US. Overall, this move not only supports reproductive autonomy but also aligns with broader public health goals, promising improved health outcomes and enhanced quality of life for many individuals.

## Funding

The authors received no financial support for the research, authorship, and/or publication of this article.

## CRediT authorship contribution statement

**Gia Tran:** Writing – review & editing, Writing – original draft, Conceptualization. **Joshua Wollen:** Writing – review & editing. **Shantera Davis:** Writing – review & editing. **Elisabeth M. Wang:** Writing – review & editing. **Julia Arriazola:** Writing – review & editing. **Natalie Rosario:** Writing – review & editing, Writing – original draft, Supervision, Conceptualization.

## Declaration of competing interest

The authors declare that they have no known competing financial interests or personal relationships that could have appeared to influence the work reported in this paper. The authors declared no potential conflicts of interest with respect to the research, authorship, and/or publication of this article.
